# Phenotypic Features and Salivary Parameters in Patients with Ectodermal Dysplasia: Report of Three Cases

**DOI:** 10.1155/2018/2409212

**Published:** 2018-03-20

**Authors:** Mônica Fernandes Gomes, Luigi Giovanni Bernardo Sichi, Lilian Chrystiane Giannasi, José Benedito Oliveira Amorim, João Carlos da Rocha, Cristiane Yumi Koga-Ito, Miguel Angel Castillo Salgado

**Affiliations:** ^1^Center of Biosciences Applied to Patients with Special Health Care Needs (CEPAPE), Institute of Science and Technology, São Jose dos Campos Campus, São Paulo State University–UNESP, São Paulo, SP, Brazil; ^2^Dental School, Metropolitan University of Santos (UNIMES), Santos, SP, Brazil; ^3^Sleep Disorders Laboratory, Universitary Center of Anápolis (UniEvangelica), Anápolis, GO, Brazil

## Abstract

Ectodermal dysplasia (ED) is a rare hereditary disorder affecting the development of ectoderm-derived organs and tissues. The aim of this study was to describe phenotypic features and the therapeutic approach in dentistry among three patients with ED, correlating their data with the literature. Additionally, to investigate the salivary gland disorders and their impacts on oral microbiota, we performed salivary tests, including salivary flow rate, salivary buffering capacity, and concentration levels of mutans streptococci, lactobacilli, and yeasts. All patients presented oligodontia, resulting in a significant masticatory dysfunction and aesthetic impairment. The counts of mutans streptococci (*n*=3) and yeasts (*n*=2) were high; on the other hand, the count of lactobacilli (*n*=3) was low. Therefore, salivary and microbiological tests showed that the patients with ED, particularly the hypohidrotic type, presented a high risk of enamel caries and susceptibility to oral infections, which may be likely triggered by reduction of salivary flow and/or possible immunological disorders.

## 1. Introduction

Ectodermal dysplasia (ED) is a rare nonprogressive congenital hereditary disorder, characterized by developmental defects of ectoderm-derived organs and tissues, affecting at least two of the following structures: nails, teeth, skin, and secretory organs (eccrine sweat, salivary, lacrimal, and mucous glands of the respiratory and gastrointestinal tracts) [1–3]. The classification of ED is based on genetic findings and phenotypic features, which is divided into two categories: anhidrotic/hypohidrotic (HED; X-linked inheritance) and hidrotic (HidED; autosomal-dominant inheritance) [4–6]. HED is the most frequent form, caused mainly by mutations in the *EDA* gene, located at the long arm of the X-chromosome (Xq12-q13.1), followed by the *EDAR* and *EDARADD* genes [4,7–10]. These genes regulate specific protein expression, especially the ectodysplasin A, which plays an important role during embryonic development [10, 11]. The HED triad includes sparse hair (hypotrichosis), reduced ability to sweat (hypohidrosis), and the lack of several teeth (hypodontia or oligodontia). Thus, the “classical” clinical characteristics are sparseness or absence of hair, eyebrows, and eyelashes; hypoplasia or agenesis of sweat, submucous, and sebaceous glands leading to episodes of heat intolerance and hyperpyrexia; dry mouth; and dentition abnormalities (incorrect numbers and shape), resulting in impaired mastication, speech disorders, and often affecting the aesthetics [4, 8, 10, 12]. Other relevant symptoms are low tear secretion, poorly functioning mucous membranes, recurrent upper respiratory tract infections, hearing or vision deficits, cleft lip and/or palate, immune dysfunction, sensitivity to light, and lack of breast development [13, 14]. HidED is caused by mutations in the *GJB6* gene, located on chromosome 13 (locus 13q12), which encodes connexin-30, a component of intercellular gap junctions. The main clinical characteristics are hair loss, palmoplantar keratoderma, dystrophic nails, atrichia or hypotrichosis, and discrete skin hyperpigmentation. Other manifestations may be found, including strabism, conjunctivitis, pterygium, cataracts, sensorineural deafness, polydactyly, and syndactyly [4, 8]. Hypoplastic submandibular glands and abnormal development of minor salivary glands have also been described [7, 13].

Based on this, we describe the phenotypic features and the therapeutic approach in dentistry among three patients with ED, correlating their data with data from literature. We also analyzed their salivary characteristics, such as salivary flow rate, buffering capacity of saliva, and concentration levels of mutans streptococci, lactobacilli, and yeasts, aiming for a better understanding of salivary gland disorders and their impacts on oral microbiota with pathogenic properties. This work was undertaken in accordance with the ethical standards of the Declaration of Helsinki.

## 2. Case Reports

### 2.1. Clinical and Radiographic Features

#### 2.1.1. Patient 1

**Figure 1:**
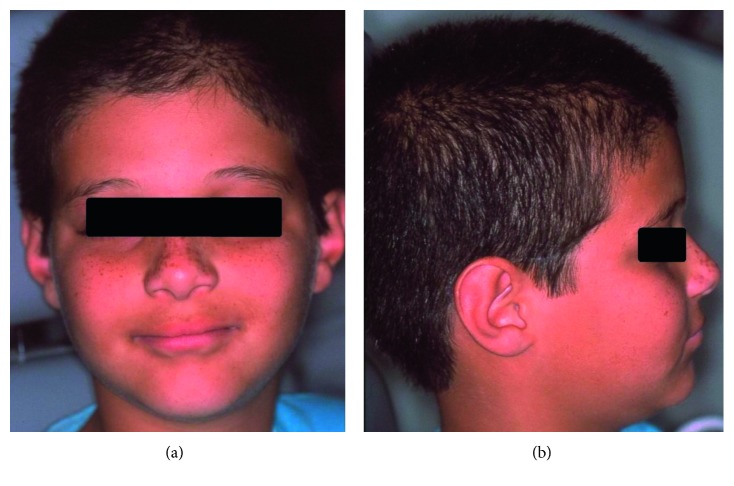
Patient 1. Child with hidrotic ED had sparse hair and eyebrows and discrete perioral pigmentation (a and b).

**Figure 2:**
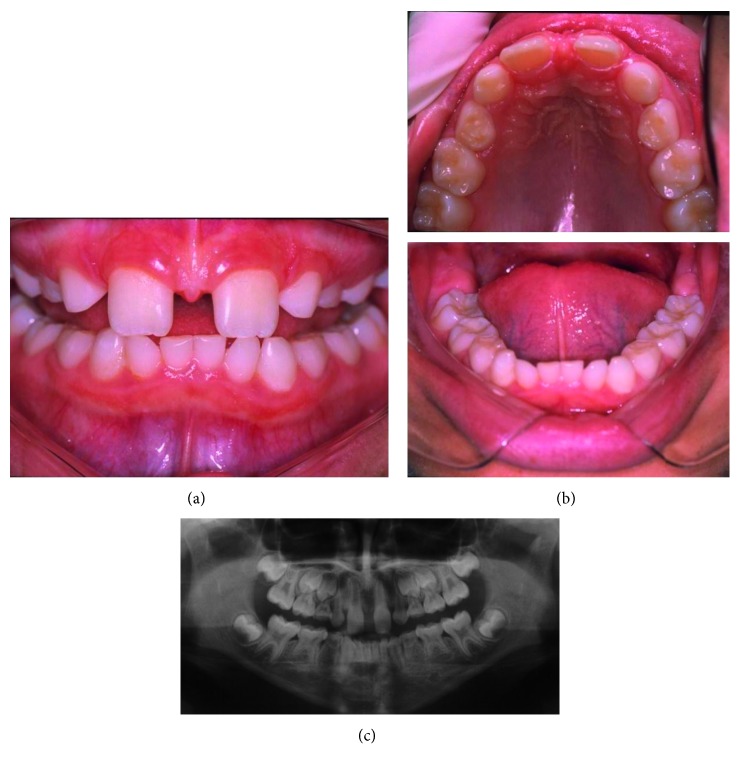
Patient 1. Accentuated diastema between the upper central incisors due to insertion of the labial frenulum (a), mixed dentition with no caries (b), and absence of the 31, 41, 12, 22, 32, 42, 13, 23, 33, 43, 34, 44, 35, and 45 teeth (c).

**Figure 3:**
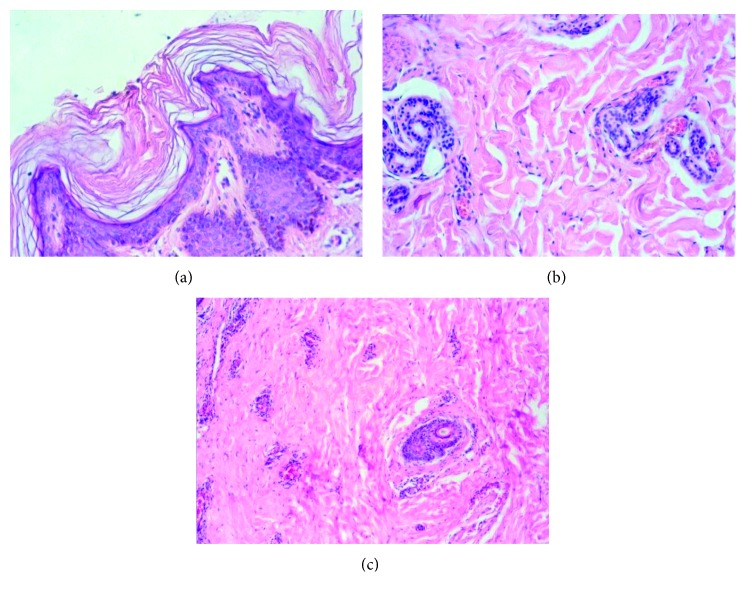
Patient 1. Histological sections showing hyperkeratosis (a), atresia of sweat glands and absence of sebaceous glands (b), and atrophic hair follicles (c) (original magnification: ×200 and ×100; hematoxylin and eosin).

An 11-year-old Caucasian male child complained of oral aesthetic impairment caused by the accentuated diastema. The mother reported delayed eruption of the primary dentition during his childhood. He had good general health; however, episodes of hyperpyrexia were reported, mainly during sports activities. Upon extraoral examination, perioral pigmentation and dry skin were evidenced (Figure 1). Upon intraoral examination, an accentuated diastema between the upper central incisors was observed due to insertion of the labial frenulum into the gum ridge. Fourteen permanent teeth were absent, including the right left lower central incisors, right left upper and lower lateral incisors, right left upper and lower canines, and right left lower second first premolars (Figure 2). Discrete mild chronic gingivitis due to the presence of supragingival dental biofilm, and no caries were also observed. Thus, an incisional biopsy in the gluteal region was performed to confirm the diagnosis of ectodermal dysplasia, and the histological sections showed few hair follicles and eccrine sweat glands and absence of sebaceous glands (Figure 3). These findings confirmed the diagnosis of HidED. The recommended treatment was dental prophylaxis, topical application of 1.23% fluoride, oral hygiene control, and occlusal adjustment of the preexisting teeth to prevent root resorptions, particularly the deciduous teeth.

#### 2.1.2. Patient 2

**Figure 4:**
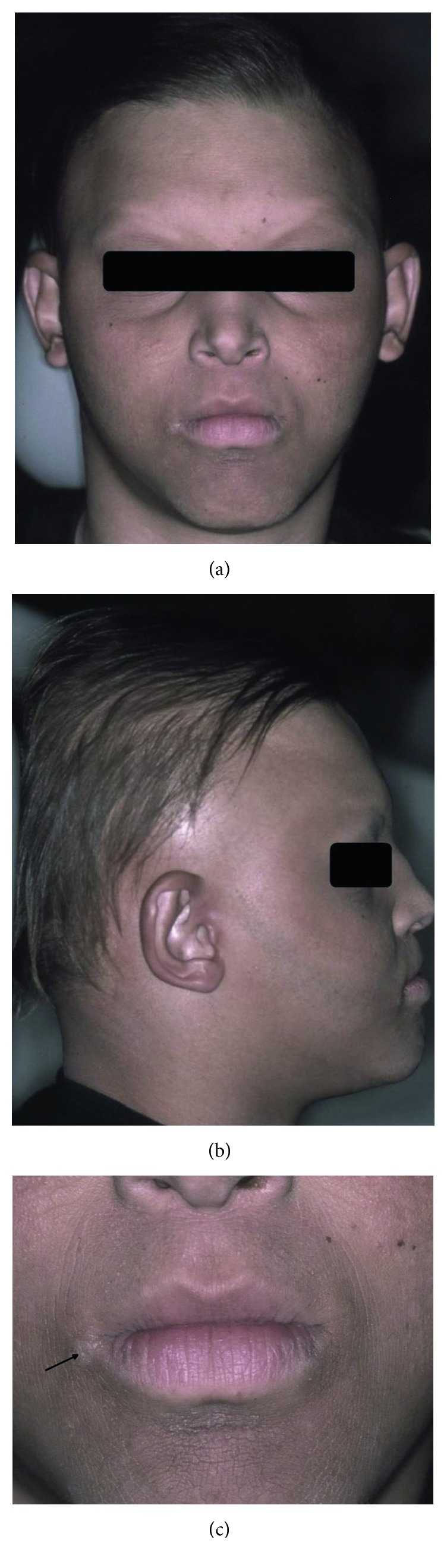
Patient 2. Boy with hypohidrotic ED displaying scarce, fine, and silky hair, alopecia of the eyebrows, prominent frontal bossing, discrete deformity and low implantation of the ear (a and b), angular cheilitis (arrow), dry and scaly skin, and perioral dermatitis and fissures (c).

A 14-year-old Caucasian adolescent boy with HED had partial anodontia and difficulties in eating, swallowing, and speech, significantly affecting his self-esteem. The medical history reported by the patient was heat intolerance and suffering from hyperpyrexia, frequent colds, and otitis, and recurrent respiratory tract infections were described. Moreover, eye drops and nasal lubricants have been frequently used to relieve eye and nose dryness, respectively. Upon general examination, dry skin and hyperkeratosis, especially in the joints of the upper and lower limbs, were described. Upon extraoral examination, scarce, fine and silky hair, alopecia of the eyelashes and eyebrows, prominent frontal bossing, discrete bilateral deformity and low implantation of the ear, perioral dermatitis and fissures, bilateral angular cheilitis, periorbital pigmentation, and deep nasolabial sulcus were evidenced (Figure 4). Upon intraoral examination, it was verified that almost all permanent teeth were absent, except the right upper permanent canine, resulting in a significant loss of occlusal vertical dimension and an appearance of old age. The upper and lower alveolar ridges were underdeveloped, and the buccal mucosa was found pale and sharply dry. Chronic mouth breathing was also diagnosed.

**Figure 5:**
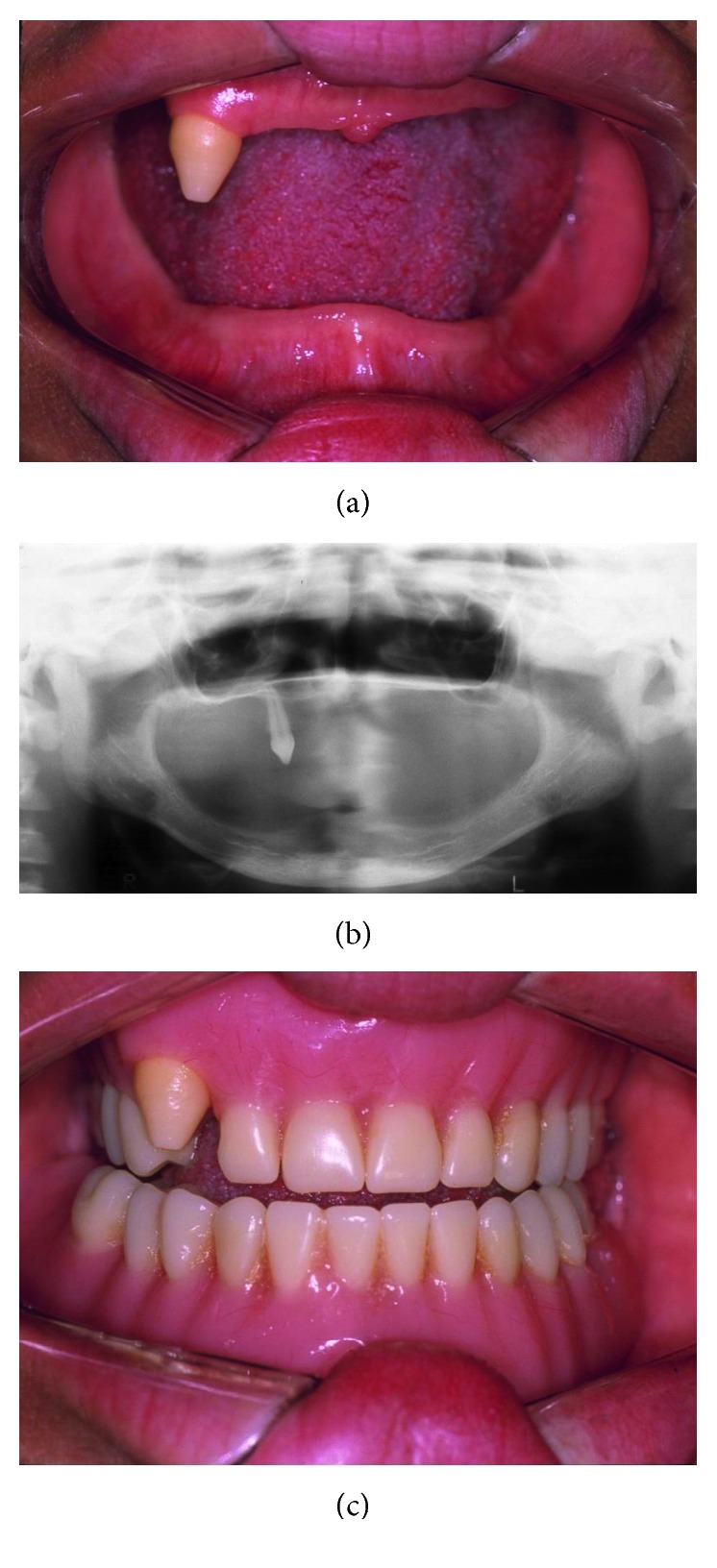
Patient 2. Agenesis of almost all permanent teeth, except the 13th tooth, underdeveloped alveolar ridge with “knife-edge” shape (a and b) and prosthodontic rehabilitation with the use of upper and lower dentures (c).

Initially, the recommended treatment was to make provisional upper and lower dentures (Figure 5), restoring the aesthetics and masticatory functions and, as a consequence, improving the patient's facial appearance and psychological well being. Concomitantly, speech therapy was also accomplished to improve the orofacial motricity after the insertion of the dentures, resulting in rejuvenation with the smoothing of wrinkles and furrows and a balance of orofacial and masticatory muscles. It is important to emphasize that this first phase of treatment was very important to establish the self-esteem of this patient, resulting in his favorable behavior change and, consequently, immediate social inclusion.

After the adaptability of the patient to the provisional dentures, mini dental implants will be inserted into the atrophic alveolar ridges of the edentulous mandible and maxillae in order to effectively retain overdentures and, thus, to promote a better stability for the new removable dentures. This treatment alternative is recommended as the patient's alveolar processes display very thin thickness and reduced height. We believe that this protocol can improve the mastication, comfort, satisfaction, and oral health-related quality of life of this individual.

#### 2.1.3. Patient 3

**Figure 6:**
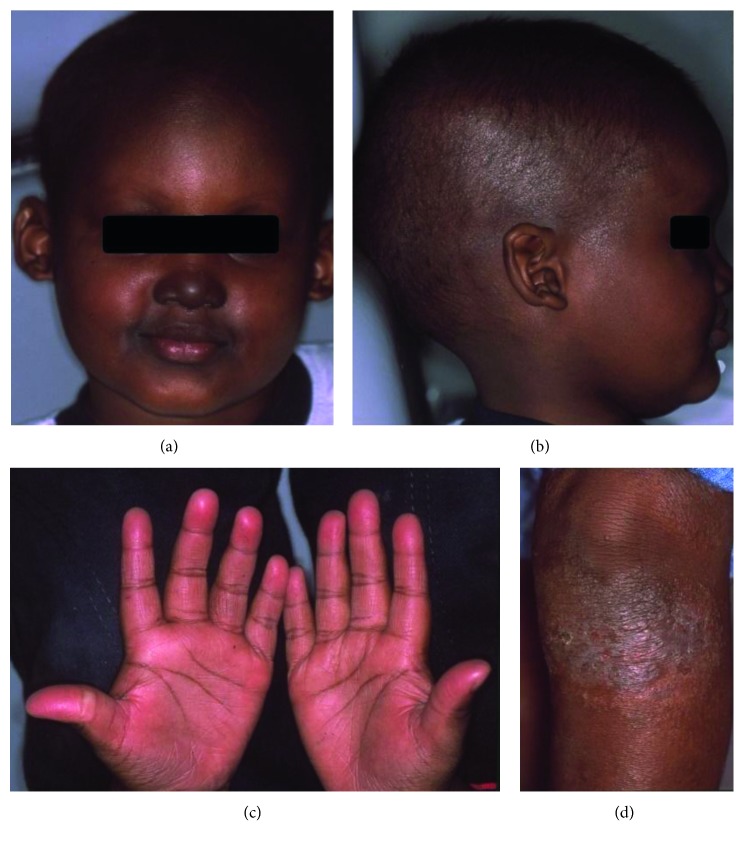
Patient 3. Child with hypohidrotic ED presented sparse hair, perioral and nasal pigmentation, ear deformity with pointed shape (a and b), and hyperkeratosis on the palms and knee (c and d).

**Figure 7:**
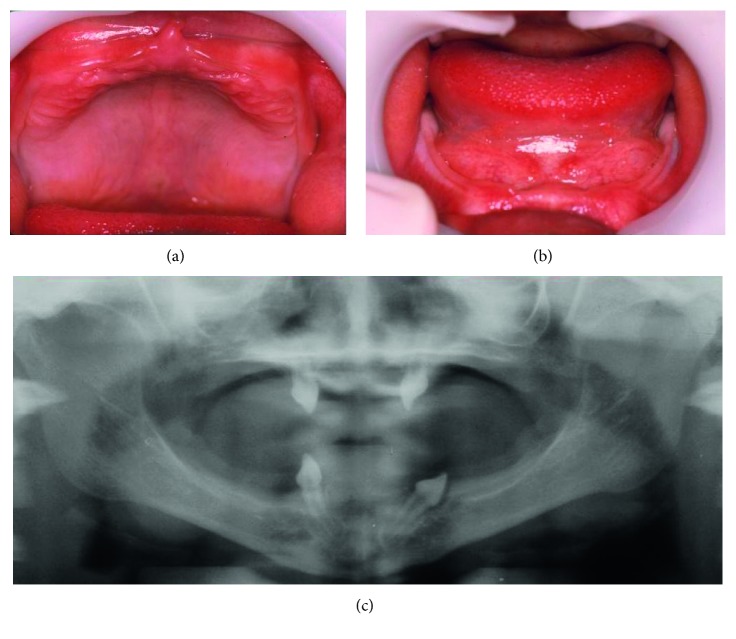
Patient 3. (a) Upper and (b) lower alveolar ridges were edentulous and underdeveloped, and (c) radiographic images showing the presence of the 53rd, 63rd, 73rd, and 83rd teeth.

A 3-year-old afrodescendent male child with HED showed great difficulty eating, swallowing, and speech. The medical history reported by the patient was hyperthermic episodes without association with focal infections, reduction of lacrimal secretion, recurrent otitis and colds, and use of antiallergic medication. Upon general examination, the facial skin presented finely wrinkled and dry skin, appearing prematurely aged. Hyperkeratosis was also observed in the elbows, knees, and ankles. Upon extraoral examination, a depressed nasal bridge (“saddle nose”), thin and scanty hair, alopecia of the eyelashes and eyebrows, dry and crusted eyes, periorbital, perioral, and nasal pigmentation, bilateral deformity and low implantation of the ear, perioral fissures, and deep nasolabial sulcus were evidenced (Figure 6). Upon intraoral examination, dry mouth was observed and no deciduous teeth were erupted; however, the radiographic images revealed the presence of the right and left lower and upper deciduous canines and the absence of all permanent dental germs. The upper and lower alveolar ridges were underdeveloped (Figure 7). Firstly, speech therapy was recommended to improve the stomatognathic system functions and to stimulate the growth of the jaw bones. Following this, provisional prostheses will continue to be made until the complete development of the mandible and maxilla, after the eruption of the preexisting deciduous teeth.

### 2.2. Microbiological and Salivary Tests

Initially, saliva was stimulated by using sugarless gum, and then the samples were collected into a sterile cup for 15 min, between 8 and 10 a.m., to prevent circadian rhythm variation. The first saliva sample was discarded to guarantee the fidelity of the results of microbiological analysis. Following this, salivary quantitative and qualitative tests were performed, including salivary flow determination, buffering capacity of saliva, and counts of mutans streptococci (MS), lactobacilli, and yeasts. The methodology applied is described in the study of Koga-Ito et al. [15], and the results obtained are demonstrated in Table 1.

## 3. Discussion

Due to craniofacial morphological anomalies, the patients with ED often present low self-esteem, psychological pressure, and limited social interactions. The dysmorphic features of the maxillofacial region and agenesis of salivary and sweat glands can lead to systemic and oral disorders [3]. Among the two types of ED, HED is more prevalent than HidED [5], being in agreement with our studies.

The ability to perspire is reduced due to sweat gland dysfunction, and so patients are predisposed to develop hyperpyrexia due to the misregulation of the body temperature [8, 11, 16, 17]. This clinical symptom was reported by patients 1 and 2, especially during sports activities or high ambient temperatures.

Facial dysmorphy, including a prominent forehead, a depressed nasal bridge, and thick lips, was also noted in patients 2 and 3. All patients have presented congenitally missing dentition since childhood, and consequently, severe oligodontia, leading to masticatory dysfunction and aesthetic impairment. Thus, as a result of several agenesis of permanent and/or deciduous teeth and narrow upper and lower alveolar ridges, the vertical dimension of the face was reduced and the lips became protuberant. These findings were evidenced in patients 2 and 3. Moreover, no dental dysmorphy was found in our patients.

Unfortunately, congenital defects involving oral cavity and facial appearance led to severe masticatory dysfunction and psychosocial disorders negatively influencing the mental health, respectively, especially for the adolescent individuals with ED. Therefore, immediate oral rehabilitation for stabilizing the aesthetics and masticatory functions must be performed, that is, using dental-mini implants due to the extensive bone hypotrophy of the alveolar processes [3]. Some studies report that implant placement in children with ED is highly debatable [3]; on the other hand, others reinforce the use of mini dental implants in children with ED, ensuring better aesthetics and functional and psychosocial development [18]. Thus, treatment strategy should include a comprehensive consideration of patient-specific aspects in order to ensure the best outcomes [3]; however, others reinforce the use of mini dental implants in children with ED, ensuring better aesthetics and functional and psychosocial development [18]. In this study, we can recommend the placement of mini dental implants to better retention of removable dentures only for the adolescent boy (patient 2).

A relevant clinical symptom was a persistent feeling of dry mouth in both patients 2 and 3; however, xerostomia was proved only in patient 3. Probably, this condition caused masticatory, swallowing, and speech difficulties. Our study has some limitations because anatomical and functional abnormalities of salivary glands were not properly investigated, using clinical methods, in particular, sialometry. Some authors described that salivary gland aplasia may lead to variable dysfunctions, including reduction on the salivary flow rate and alterations on the salivary composition [13, 19]. The oral mucosa becomes dry and atrophic, and the patients can gradually show dysgeusia, dysphagia, and dysarthria, as well as risk of developing ulcerations, caries, gingivitis, periodontitis, candidosis, and bacterial sialadenitis, among others [19]. We suggested that adaptive or assistive technology should be recommended for patients with hyposalivation and dysphagia, as support therapies of gustatory and neuromuscular mechanical stimulation, in order to strengthen the muscular tone, in particular, the masticatory muscles, and to increase the production of saliva.

In this study, our patients were more susceptible to oral infections by bacteria and yeasts. Additionally, risk of malnutrition due to dysphagia and difficulties of mastication and speech may be found, resulting in an important harm to the oral homeostasis and to the quality of life. Another relevant feature is the appearance of rampant caries and severe periodontal disease, resulting in extensive damage of the buffering and antimicrobial properties of saliva [13].

Concerning the immunodeficiency disorder in ED, we consider that patient 2 was immunosuppressed once the diagnosis of angular cheilitis caused by *Candida albicans* was confirmed. Furthermore, recurrent symptoms of colds, otitis, and respiratory tract infections were also reported. Some studies reported that individuals with ED can present abnormalities in the immune system, as protein deficiency of the nuclear factor kappa-*β* essential modulator (NEMO). This protein is encoded by the *IKBKG* gene. This mutation causes impaired cytotoxicity mediated by natural killer cells and impaired CD40 signaling with resultant hypogammaglobulinemia, decreased antibody response to polysaccharide antigens, and elevated IgM levels. Thus, these factors may impair the patient's defense mechanisms against pathogens, favoring the development of diseases [2, 14]. Although investigations of immune system phenotyping and explorations of NEMO gene mutations were not performed in our patients, we advocate the importance of these specialized biological analyses to confirm the immunodeficiency disorder and to identify its degree of severity, especially in patients with ED.

Regarding the buffering capacity of saliva, no significant alteration was detected in our patients; however, patient 3 was instructed to ingest only basic foods due to xerostomia. According to Chifor et al. [20], the buffering capacity of saliva allows neutralization of plaque acids and remineralization of early enamel caries lesions, leading to a protective effect for potentially pathogenic microorganisms.

Although caries etiology is understood as a polymicrobial and tissue-dependent disease, mutans streptococci are considered one of the most relevant etiologic agents involved in the acidogenic stage [21, 22]. For this reason, counts of mutan streptococci have been used as caries risk indicator or evaluation of anticaries therapy [15, 23]. Besides, counts of lactobacilli are also widely used for caries risk prediction. Lactobacilli are frequently found in low pH areas of caries lesions and exhibit acidogenic ability [24, 25].

It is important to highlight that *Candida* species are associated with caries etiology once they are able to form considerable quantities of acid from carbohydrates, leading to a decrease of the salivary pH [26]. Moreover, *Candida albicans* is also seen as an opportunistic microorganism that may trigger an oral lesion, especially in immunosuppressed individuals [27].

In our study, the counts of mutans streptococci (*n*=3) and yeasts (*n*=2) were high; in contrast, the count of lactobacilli was low. These findings show that our patients presented great predisposition to enamel caries and opportunistic oral infections. It is important to highlight that, although patient 2 has presented no caries risk to yeasts, candidiasis was confirmed bilaterally in the oral commissure. This illness, probably, occurred due to the favorable biological environment promoted by the accentuated loss of vertical dimension, plus the susceptibility of the patient to infections. Therefore, the buccal prophylaxis and orientation of oral hygiene must be indicated as a supportive treatment, especially for the dentulous patient (patient 1) due to predisposition to caries.

Oral cavity is considered a gateway and a reservoir for pathogenic microorganisms, especially in immuno suppressed patients [14]. Considering this, more research related to protein composition of saliva may be performed since the salivary antimicrobial proteins or circulating immune complexes, containing IgA, IgG, and IgM, could be carefully investigated in individuals with ED.

## 4. Conclusion

Based on these investigations, our patients with ED, particularly the hypohidrotic type, presented a high risk of enamel caries and susceptibility to opportunistic oral infections, which may be likely triggered by reduction of salivary flow and/or possible immunological disorders. However, more investigations must be performed to elucidate the functional behaviors of oral microbiota in these individuals and to explain the appearance of oral lesions caused by biological agents.

## Figures and Tables

**Table 1:** Amount of salivary flow and buffering capacity of saliva, and counts of mutans streptococci, lactobacilli, and yeast in patients with ED.

Salivary tests	Patient 1 (HidED)	Patient 2 (HED)	Patient 3 (HED)
*Salivary flow rate (mL/min)*			
Normal flow: >1.0	1.0 (limit value)	1.2 (normal)	<0.1 (xerostomia)
Limit value: 1.0			
Reduced flow: ≤0.7			
Xerostomia: ≤0.1			
*Buffering capacity of saliva (pH value)*			
Normal: 5.1–7.0	4.0 (limit value)	6.0 (normal)	5.0 (limit value)
Limit value: 4.0–5.0			
Low buffering capacity: <4.0			
*Mutans streptococci (log·cfu/mL)*			
High caries risk: >5.0	6.2 (high)	5.0 (high)	4.9 (high)
*Lactobacilli (log·cfu/mL)*			
Low caries risk: 0.0–3.0	0.0 (low)	2.75 (low)	1.70 (low)
Moderate caries risk: 3.0–3.7			
High caries risk: >4.0			
*Yeasts (log·cfu/mL)*			
No caries risk: 0–1.0	3.0 (high)	0.0 (no caries risk)	2.78 (high)
Moderate caries risk: 2.0–2.6			
High caries risk: >2.6			

HidED, hidrotic ectodermal dysplasia; HED, hypohidrotic ectodermal dysplasia.
